# Xenotransplantation Research -the Nonhuman Primate Model Is Preferable to the Human Decedent Model

**DOI:** 10.3389/ti.2025.14452

**Published:** 2025-08-07

**Authors:** D. K. C. Cooper, L. Mou, J. D. Cleveland, J. H. Simmons, D. C. Cleveland

**Affiliations:** ^1^ Department of Surgery, Center for Transplantation Sciences, Massachusetts General Hospital/Harvard Medical School, Boston, MA, United States; ^2^ Shenzhen Xenotransplantation Medical Engineering Research and Development Center, Shenzhen Institute of Translational Medicine, Health Science Center, The First Affiliated Hospital of Shenzhen University, Shenzhen Second People’s Hospital, Shenzhen, China; ^3^ Children’s Hospital of Los Angeles, University of Southern California, Los Angeles, CA, United States; ^4^ Michale E. Keeling Center for Comparative Medicine and Research, University of Texas MD Anderson Cancer Center, Bastrop, TX, United States

**Keywords:** kidney, xenotransplantation, brain death, decedent, nonhuman primate

## Abstract

Over the past 40 years, the pig-to-nonhuman primate organ transplantation model has enabled progress in xenotransplantation to be made to the point that we are now carrying out initial US FDA-approved clinical experiments on “compassionate” grounds. More recently, the pig-to-human brain-dead decedent model was introduced with claims that this might replace (or at least augment) the pig-to-NHP model. There are, however, several limitations of the decedent model, most notably the very limited period during which the subject may remain sufficiently metabolically and hemodynamically stable to allow meaningful monitoring of the fate of a pig organ graft. It will be exceedingly difficult to provide the regulatory authorities with data from experiments in which truly prolonged graft function has been monitored, whereas this is already being achieved in the pig-to-NHP model. In view of the complications related to the effects of brain death, the data obtained from xenotransplantation experiments in decedents may provide confusing results. There is a real risk that this may influence the regulatory authorities to become overly cautious in approving formal clinical trials of pig organ xenotransplantation to be initiated. We conclude that experiments in human decedents will be unable to replace studies in pig-to-NHP models.

## Introduction

The pig-to-nonhuman primate (NHP) model has formed the basis for xenotransplantation research for the past 40 years [1]. It is this model that has advanced xenotransplantation to the point today where we have been able to initiate clinical experiments. Without the data accumulated from this model over the past 40 years, little or no progress would have been made. However, there are some researchers today who are disparaging of this model. Furthermore, some of those who are most critical of the model and have introduced the human decedent model have ignored the advances made in the NHP model during the past 20 years. We provide examples below.

When comparing the past and future contributions of the two models, a very important consideration is what information and data the regulatory authorities, e.g., the Food and Drug Administration (FDA) in the USA, require before they approve of formal clinical trials of xenotransplantation. With regard to pig kidney xenotransplantation, to our knowledge the US FDA requires evidence of “consistent” survival of pig kidneys transplanted into primates for 12 months. By “consistent,” it is uncertain what will be required, but we anticipate that they mean perhaps 6 of 8 consecutive pig kidney grafts being life-supporting for 12 months. Furthermore, the FDA ideally requires that the kidneys should be of the exact pig genotype and the immunosuppressive therapy administered should be identical to that which will be used in the proposed clinical trial. Given the differences in the immune response between NHPs and humans to certain gene-edited pig kidneys, e.g., those from triple-knockout (TKO) pigs [2, 3], the FDA may allow some differences if there is good evidence justifying these.

In view of the challenges of maintaining immunosuppressed NHPs alive and well for a period of 12 months under laboratory conditions (that are primitive in comparison with those in a modern hospital), this will prove challenging. Although the number of NHPs with a pig kidney that have survived for periods >12 months is steadily increasing [4, 5], Kinoshita K, manuscript in preparation), to obtain 6 of 8 consecutive successes will remain difficult. In the light of the results of the small number of clinical experiments being conducted, the FDA may modify its requirement. However, we suggest it will be even more difficult (and expensive) to achieve this in human decedents. Furthermore, the ethics of maintaining a brain-dead human subject under experimental conditions for months may be questioned.

## The Pig-To-NHP Model

The observations made from pig-to-human *in vitro* studies (Table 1A) [6] and from pig-to-NHP studies (Table 1B) are numerous, and we suggest that, although studies in the pig-to-human decedent model have confirmed several of the observations made in pig-to-NHP models (Table 2), nothing truly new has been “discovered” in the decedent model.

**TABLE 1:** Selected studies in the pig-to-nonhuman primate (NHP) and pig-to-human models.

A: Selected *in vitro* studies in the pig-to-human or NHP model
1. Anti-wild-type (WT) pig antibodies develop during the first year of life
2. There is a correlation between IgM level (but not IgG) and complement-dependent cytotoxicity (CDC.).
3. Some adult humans have *no* natural anti-TKO pig antibodies
4. All NHPs have antibodies to TKO pig cells
5. A small percentage of allosensitized humans have antibodies to TKO pig cells
6. Deletion of Gal is associated with a reduced human T cell response
B: Selected *in vivo* studies in the pig-to-NHP model
1. Wild-type (WT) pig organ transplantation results in hyperacute rejection; GTKO/TKO pig organ transplantation does not result in hyperacute rejection
2. Expression of a human complement-regulatory and/or coagulation-regulatory protein extends pig graft survival
3. A systemic inflammatory response has been documented
4. Sensitization to pig antigens is *not* detrimental to subsequent allotransplantation
5. CD40/CD154 co-stimulation pathway blockade is superior to conventional therapy, e.g., tacrolimus-based, in an immunosuppressive regimen
6. After pig kidney transplantation, pig renal function has some differences from native NHP renal function

**TABLE 2:** Summary of results of pig organ transplantation into human decedents.

Experiment	Organ	Pig Genetics	Immunosuppression	Outcome	What was learned
NYU Case 1	Kidney	GTKO	Reduced conventional	No hyperacute rejection, good urine output, inconclusive function	Confirmed no hyperacute rejection with GTKO pigs
NYU Case 2	Kidney	GTKO	Reduced conventional	No hyperacute rejection, good urine output, focal C4d staining	Confirmed no hyperacute rejection; possible early rejection signs
UAB Case 1	Kidney	10-gene	Conventional	Poor function, TMA, coagulopathy	Confusing results; unclear if due to brain death or immune response
UAB Case 2	Kidney	10-gene	Conventional	Good function, excessive urine output	Pig kidneys can function but with potential complications
NYU Heart 1	Heart	10-gene	Reduced conventional + eculizumab	Successful function for 66 h	Pig hearts can function short-term in decedents
NYU Heart 2	Heart	10-gene	Reduced conventional + eculizumab	Deteriorating function	Variability in outcomes; possible immune response

Attention has been drawn to the differences in the immune response to TKO pig grafts between humans and NHPs that are related at least in part to the fact that Old World NHPs, like wild-type (i.e., genetically-unmodified) pigs, express the carbohydrate N-glycolylneuraminic acid (Neu5Gc), whereas humans do not [2, 3]. Deletion of expression of Neu5Gc in the pig, as occurs in TKO pigs, appears to result in the expression of another carbohydrate (hitherto unknown) against which NHPs have natural antibodies and strong complement activity [3].

This difference has been put forward by those advocating the human decedent model as a reason why the pig-to-NHP model may not be ideal and does not faithfully mimic the pig-to-human model. However, this major disadvantage of the NHP model has been largely overcome in NHP recipients by (i) selecting NHPs with low anti-TKO pig antibody levels and (ii) immunosuppressing with a CD40/CD154 co-stimulation blockade-based regimen (see below). The fact that pig kidney and heart transplantation in such “sensitized” NHPs can be successful despite this disadvantage surely strengthens the arguments in favor of this model (because there will be many human potential recipients with low levels of anti-TKO pig antibodies [6, 7]).

## The Pig-To-Human Decedent Model

This model is limited by several factors [8], of which three are perhaps most important. (i) The short period of time that it may be possible to monitor a pig graft in a stable decedent. This is because brain-dead subjects can become metabolically and hemodynamically unstable, particularly if there is a prolonged period of time between the onset of brain death and the insertion of a xenograft, that may affect graft function and therefore make it difficult to monitor this function accurately. A truly longitudinal study may prove impossible. (ii) There is evidence of a major inflammatory response to brain death that can exacerbate rejection and be detrimental to the induction of immunological tolerance. (iii) In addition, the severe systemic hypertension and cytokine “storm” that can accompany the onset of brain death can result in structural injury and/or cell infiltration in vital organs. At best, the brain-dead model represents a suboptimal environment to assess the response to a pig xenograft.

This can be exemplified by several of the experiments in decedents that have been reported (Table 3). The design of some of these was less than ideal, and this undoubtedly contributed to the confusing results that were obtained, making it difficult to determine whether the result was related to the effects of brain death or to the presence of a pig xenograft [9, 10].

**TABLE 3:** Selected confusing factors in some of the initial experiments in human decedents.^a^.

1. Native kidneys were *not* excised (thus making it difficult to ascertain pig kidney graft function)
2. Native kidneys were *not* studied (as ‘controls’ that had been subjected to the effects of brain death)
3. Kidneys were from GTKO pigs (rather than from pigs with multiple beneficial gene edits)
4. A conventional immunosuppressive regimen was administered (rather than a proven co-stimulation blockade-based regimen)
5. Autologous pig thymic tissue was placed under the kidney capsule. (Did it influence the outcome in any way?)
6. Consumptive coagulopathy developed, and the experiment was terminated for ‘exsanguinating hemorrhage’. (was this a consequence of brain death or the presence of a xenograft?)

^a^ Based on Cooper and Kobayashi (2023) (Ref 14, with permission).

One observation made in one of the human decedent experiments was that the pair of pig kidneys that had been transplanted into the brain-dead recipient produced 37 L of urine in the first 24 h [11]. As, to our knowledge, such a massive diuresis has not been recorded in the pig-to-NHP model and was not seen after pig kidney transplantation into a living patient carried out at the Massachusetts General Hospital in early 2024 [12], this was almost certainly a consequence of diabetes insipidus related to brain death. However, a similar massive diuresis was recorded when chimpanzee kidneys were transplanted into living human patients some 60 years ago, and one patient actually died because fluid replacement could not keep up with the urine output [13]. This type of experience might confuse the regulatory authorities as to the safety of clinical pig kidney xenotransplantation.

In particular, two decisions made by some of the investigators rendered the results much less valuable than they could have been [14].

**FIGURE 1:**
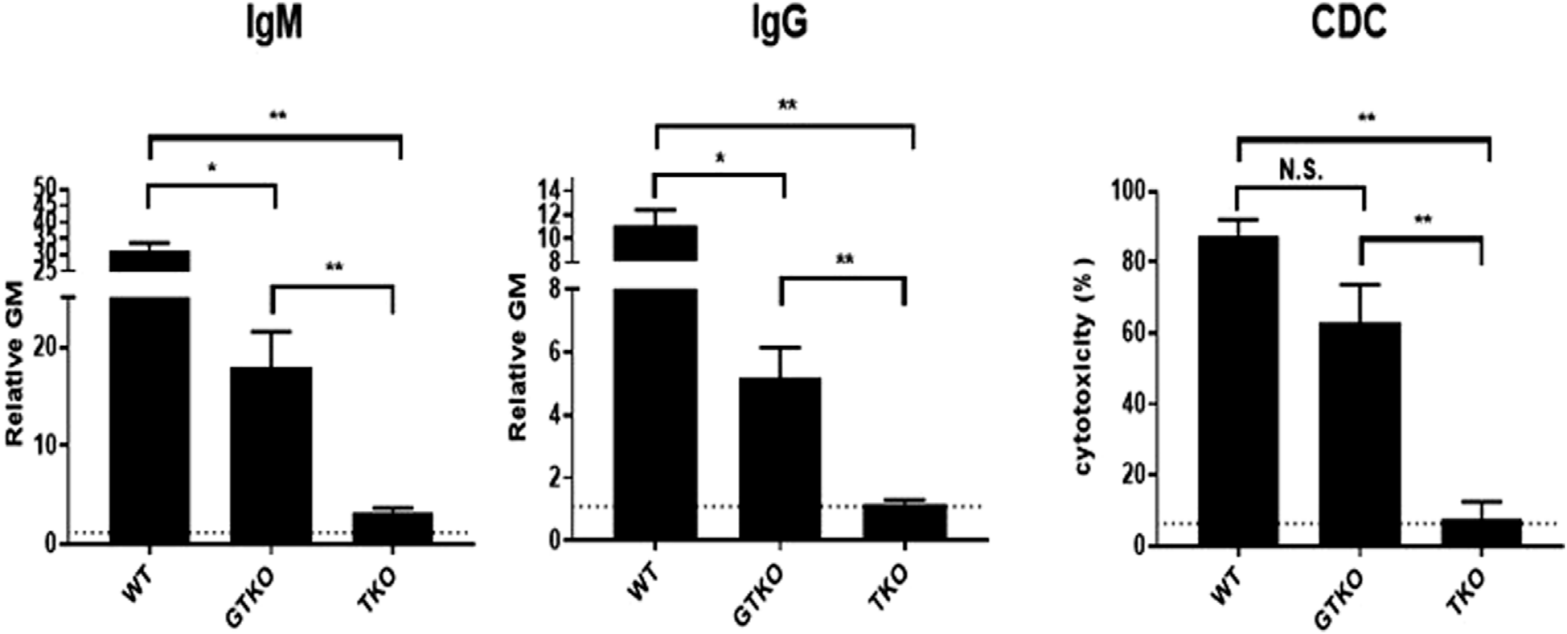
Human serum natural IgM and IgG antibody binding and complement-dependent cytotoxicity (CDC) to wild-type (WT), GTKO, and TKO pig peripheral blood mononuclear cells. The greatly reduced antibody binding and CDC to TKO cells is obvious. (Source: Hara H, et al, 2022, Ref 6, with permission).

One was to transplant an organ from a pig with only a single gene edit (α1,3-galactosyltrabnsferase gene knockout, GTKO), a pig that was state-of-the-art in 2004 (20 years ago) but has subsequently been superseded by numerous pigs with multiple gene-edits that more effectively protect the pig organ from the primate immune response [15] (Figure 1). If one is serious in wishing to assess whether xenotransplantation is ready for clinical trials, why transplant an organ from a suboptimal pig?

The second was to administer a conventional immunosuppressive regimen (as administered to patients with an allograft), when all the experimental data indicate that a CD40/CD154 co-stimulation blockade-based regimen is preferable [16–18].The argument that the regulatory authorities, e.g., the US FDA, may not approve CD40/CD154 co-stimulation blockade agents for clinical trials is fallacious because the FDA (i) has already approved some of these agents for treatment of other conditions, (ii) has approved them for formal clinical trials of human kidney allotransplantation, (iii) has allowed their use in a growing number of clinical xenotransplantation experiments on “compassionate” grounds, and (iv) has stated that it is accepted that xenotransplantation is unlikely to be truly successful unless this form of therapy is utilized. It has been argued that the human decedent models enable conventional therapy to be assessed, but a study lasting only a few days (or even a few weeks) is surely inadequate on which to come to a conclusion.

A major concern is that, if the results of studies in human decedent models continue to provide confusing results, this may influence the regulatory authorities to become overly cautious in allowing clinical trials of xenotransplantation to proceed. One final question that remains unanswered is whether the immune system weakens with time after brain death has taken place, as it does in the elderly. Is it as vigorous 12 months after the induction of brain death as it is 12 days after the induction of brain death?

The only positive outcomes from decedent studies to date are (i) they confirmed that if an organ from a GTKO or multiple gene-edited pig is transplanted into a human decedent, then hyperacute rejection does not occur (as predicted by numerous *in vitro* studies dating back to 2005), and (ii) they stimulated public interest in the field of xenotransplantation, and drew further attention to its immense potential and, importantly, how close we are to realizing this potential.

## Further Examples of the Limitations of the Decedent Model

1. To fully assess the success of gene-edited pig islet or islet-kidney xenotransplantation, follow-up will be (i) required for several months, (ii) in recipients who are physically active, and (iii) who are taking a normal diet. Is a decedent, who is not moving or eating, a suitable subject for such a study?
2. Orthotopic pig heart transplantation is being explored as a “bridge” to allotransplantation in infants with complex congenital heart disease [19, 20]. Studies in young baboons by our group have demonstrated that (i) a pig heart can sustain a young baboon for >4 months (the median period of time it takes in the USA for a suitable cardiac allograft to become available for an infant) – and, indeed, on occasions for >12 months (Cleveland JD et al, unpublished), and (ii) the replacement of the pig xenograft by an allograft is successful and is unlikely to be associated with immunological problems (Cleveland JD et al. unpublished). Consider how extraordinarily difficult–if not impossible - it would be to carry out this experiment in a human decedent model.
3. Whether the induction of immunological tolerance to a xenograft is to be achieved by hematopoietic cell chimerism or thymic transplantation, it usually requires significant pre-transplant immune modulation, e.g., irradiation or chemotherapy. Can this be tested effectively in decedents when there is little time to prepare the recipient before hemodynamic instability may occur? Furthermore, it may take many weeks or months to develop tolerance, which will require a prolonged period of follow-up. Will this be possible in a decedent?

Many other examples could be given of the difficulties of carrying out these experiments in decedents. In contrast, they are relatively straightforward in the pig-to-NHP model.

There is, however, one possible topic that might usefully be explored in human decedents. The literature suggests that highly allosensitized patients who do not have antibodies that cross-react with TKO pig cell antigens would *not* be at risk of early TKO pig organ graft rejection [21]. The transplantation of a TKO pig kidney into an allosensitized, immunosuppressed human decedent (who did not have cross-reacting antibodies) might prove that this assumption is correct. The result could, of course, be compared with that of a TKO pig kidney grafted into a highly-sensitized decedent who *did* have cross-reacting antibodies.

## Comment

We suggest that we have enough data from research in NHPs to initiate a meaningful and ethical clinical trial of pig kidney transplantation today [22], and we are close to undertaking a clinical trial of pig cardiac transplantation in infants. What is now required is to carry out an increasing number of pig organ transplants in living human patients, not in human decedents, which we suggest would be a distraction. If there is a need to investigate organ transplantation from (i) pigs with novel gene edits, or (ii) in recipients who will receive an immunosuppressive regimen that includes truly novel agents or a novel tolerance-inducing regimen, this can only be satisfactorily achieved in a pig-to-NHP model (with prolonged follow-up) but *not* in a decedent model.

## Data Availability

The original contributions presented in the study are included in the article/supplementary material, further inquiries can be directed to the corresponding author.

## Abbreviations

FDA, US Food and Drug Administration; NHP, nonhuman primate; TKO, triple-knockout (pig).
